# Now You See, Now You Don’t: A Case Of Spontaneous Regression of Pituitary Tumour

**DOI:** 10.7759/cureus.9174

**Published:** 2020-07-14

**Authors:** Ainul Syahril Jaafar, Siti salwa Mohd shokri, Sanmugarajah Paramasvaran, Kamalanathan Palaniandy, Farizal Fadzil

**Affiliations:** 1 Neurosurgery, Universiti Kebangsaan Malaysia Medical Center, Kuala Lumpur, MYS; 2 Surgery, Universiti Kebangsaan Malaysia Medical Center, Kuala Lumpur, MYS; 3 Neurosurgery, Universiti Sains Malaysia, Kuala Lumpur, MYS

**Keywords:** spontaneous regression, pituitary tumour, lymphocytic hypophysitis, hypothyroidism, pituitary apoplexy

## Abstract

Spontaneous regression of pituitary tumours are rare and can be due to tumour ischaemia, pituitary apoplexy, or lymphocytic hypophysitis. We report a case of a 32-year-old female, who presented with symptoms and signs of extrasellar pituitary enlargement and hypothyroidism. MRI revealed a pituitary mass that spontaneously regressed after a month, with complete resolution of symptoms. Not all pituitary tumours require surgical intervention especially in the case of autoimmune lymphocytic hypophysitis.

## Introduction

Pituitary tumours are common intracranial neoplasms, they account for 10% to 15% of all intracranial tumours of which 90% of them are adenomas [1]. Most macroadenomas are non-functioning with half of the cases will grow, while another 11% will spontaneously regress [2]. Spontaneous regression of pituitary tumour can be due to tumour ischaemia, pituitary apoplexy or lymphocytic hypophysitis [2,3]. In general, pituitary tumours are treated surgically in patients with visual impairment or endocrinopathies. In patients not surgically treated, regular follow-up is required to evaluate tumour growth as well as visual and endocrine functions.

## Case presentation

**Figure 1:**
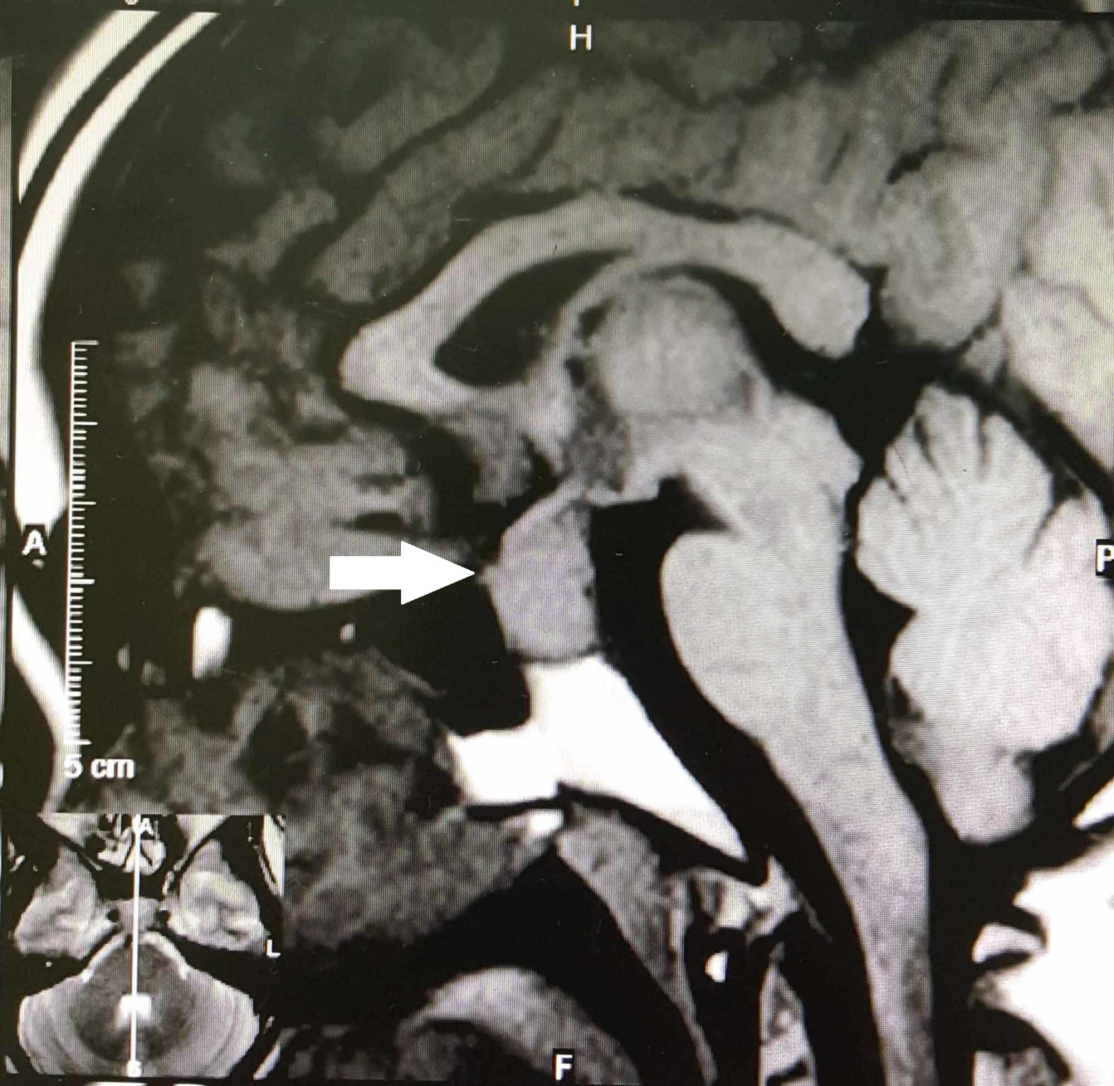
T1-weighted sagittal MRI showing a pituitary mass with thickened stalk

A 32-year-old female presented with three months history of headache, amenorrhea, and lethargy, followed by worsening of nausea and vomiting. Apart from that, there was no visual defect, weight loss, or symptoms suggestive of diabetes insipidus. She is single and denied any personal or family history of autoimmune disorders. Her body mass index was 23 and had no neurological deficit. Initial MRI showed a pituitary mass with thickened stalk, extending into the cavernous sinus with a small gap from optic chiasm (Figure 1).

**Figure 2:**
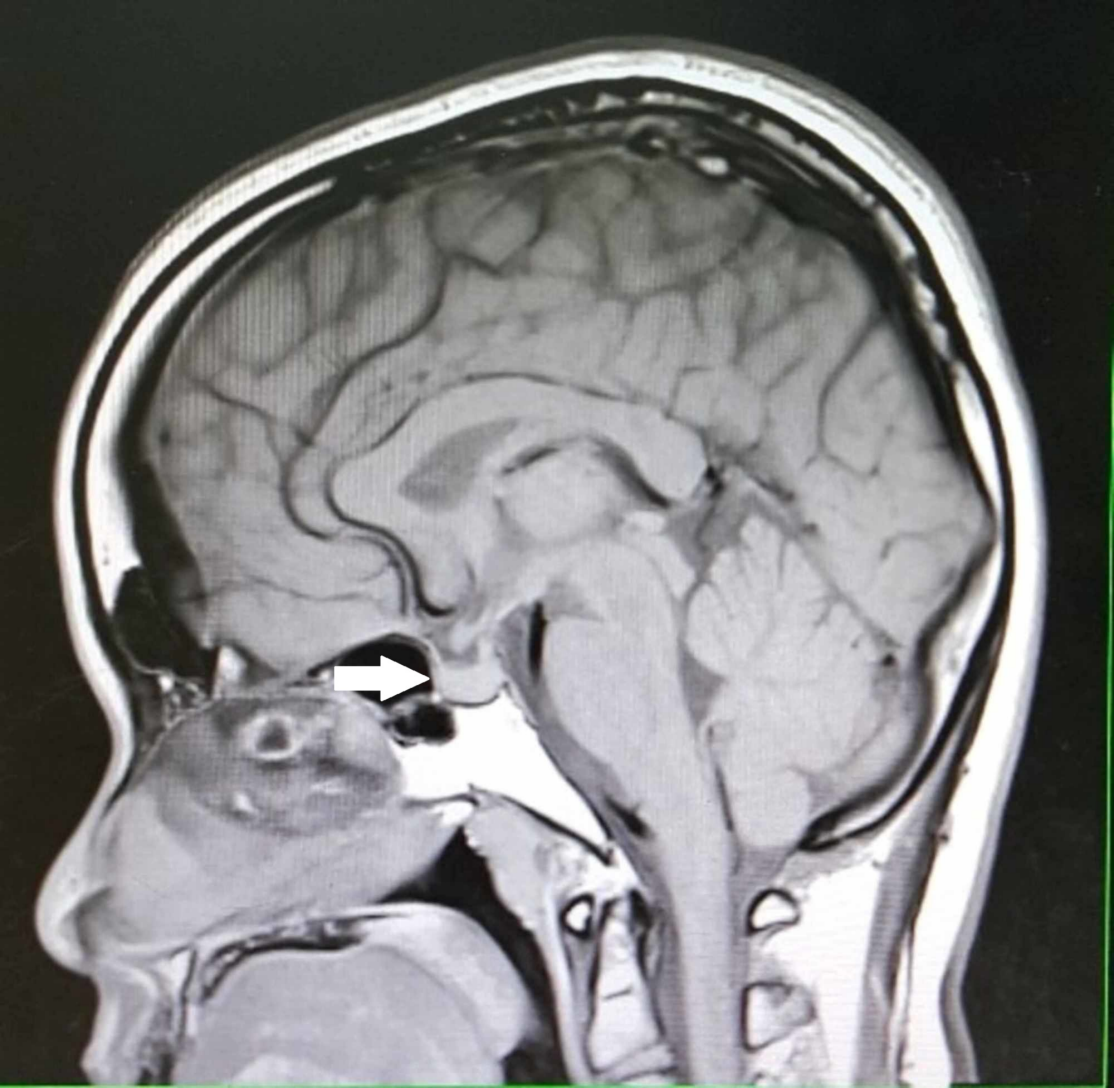
T1-weighted sagittal MRI repeated after one month revealed a shrinkage of pituitary mass

She was hypothyroid at presentation, thus started on levothyroxine 50 ug daily. Other pituitary hormone profiles were within the normal range. Liver function tests were also normal. However, her fasting lipid profiles showed high total cholesterol 8.16 mmol/L (<5.18), with low-density lipoprotein (LDL)-cholesterol of 6.24 mmol/L (<3.80) and triglycerides of 2.14 mmol/L (<1.7). She was initially referred to the neurosurgical team surgery, however, a repeat MRI one month later showed marked shrinkage of the pituitary tumour (Figure 2).

In view of tumour involution in the recent MRI, her surgery was deferred. She was then followed up by the endocrinologist and was treated as autoimmune lymphocytic hypophysitis. After three months, all her symptoms have subsided, and her menses was back to normal.

## Discussion

Patients with pituitary tumours commonly present with symptoms due to local effect of the mass secondary to pituitary enlargement and symptoms related to pituitary hormonal alterations. The natural course of these pituitary tumours is not well known because the majority of them are operated [2,4]. However, patients with stable, improving or no visual symptoms can be managed conservatively. Surgery is also indicated for local tumour control and in patients with tumour elevating the chiasm even if there are no visual defects [4].

Our patient presented with headache, which was most likely due to local effect of the mass. She was also amenorrheic, lethargic, and had hypercholesterolaemia. Her initial thyroid function test showed low serum T4 levels with elevated thyroid-stimulating hormone (TSH). Of note, hyperprolactinaemia can also cause amenorrhea, but her serum prolactin was normal. Her initial MRI showed pituitary macroadenoma and she was planned for surgery. However, a repeat MRI scan prior to surgery showed tumour shrinkage.

Lymphocytic hypophysitis is an autoimmune disease in which the pituitary gland is infiltrated by lymphocytes, plasma cells, and macrophages, causing impairment of its function. The pathogenesis is still unclear. An autoimmune pathogenesis as well as a viral origin has been suggested [3]. Prior to the MRI scan and pituitary biopsy, very few cases have been reported. It predominantly affects women, and frequently presents in the last six months of pregnancy and in the first six months after delivery [3,5]. However, there are cases reported outside pregnancy, usually affecting those with a family history or their own history of autoimmunity. Hyperprolactinemia affects one-third of patients with lymphocytic hypophysitis, causing amenorrhea and/or galactorrhea in women and sexual dysfunction in men. It can also cause thyrotropin (TSH) and/or gonadotropin deficiencies [3].

MRI scan is the best imaging diagnostic tool in differentiating lymphocytic hypophysitis from pituitary adenomas. MRI scan features suggestive of lymphocytic hypophysitis include symmetrical enlargement of the pituitary gland, thickened pituitary stalk, and an intact sellar floor. In contrast, pituitary adenomas are usually asymmetric, often displacing the infundibulum, rarely involved the stalk or erode the sellar floor [3,5]. They also appear heterogeneous both before and after contrast medium administration, though heterogenicity can also occur in lymphocytic hypophysitis. Despite the above available criteria, findings tend to overlap, and no single sign is diagnostically accurate. For this reason, histopathological findings from pituitary biopsy remain the gold standard in diagnosing lymphocytic hypophysitis which necessitates an invasive approach [3,5].

In this particular case, our patient was diagnosed with lymphocytic hypophysitis based on her clinical presentation and MRI scan features of thickened pituitary stalk which favours the diagnosis of autoimmune hypophysitis. Even though it commonly affects women in their late pregnancy or in the early postpartum period, cases occurring outside pregnancy is not unusual. It also usually affects those with autoimmune diseases. Interestingly, our patient was nulliparous and had no autoimmune disease or family history as such.

Although it is rare, pituitary apoplexy regression, which is clinically silent and radiologically undetectable tumour ischaemia, should be considered in the differential diagnosis. Pituitary apoplexy occurs when the large tumour compresses or outgrows the blood supply resulting in ischaemic necrosis and haemorrhage into the necrotic tumour [4,6,7]. It is a well-known phenomenon in cases of functioning pituitary adenomas [8]. Following apoplexy, it is believed that there is a decrease in the production of pituitary hormones by hormonally active adenomas, leading to spontaneous resolution of symptoms and in some cases, the patient can have hypopituitarism [6].

This patient was treated with oral levothyroxine for her hypothyroidism. Subsequent follow-up showed normalization of her thyroid function tests and complete resolution of her symptoms. As the pituitary gland had significantly decreased in size, surgery was no longer indicated.

## Conclusions

Autoimmune lymphocytic hypophysitis should be considered in cases of spontaneous regression of pituitary tumours. We also conclude that in the absence of visual field defects, observation alone is a safe alternative for surgery, especially in patients without compromised pituitary function and without compression of the optic chiasm.
